# Geographic strain differentiation of *Schistosoma japonicum* in the Philippines using microsatellite markers

**DOI:** 10.1371/journal.pntd.0005749

**Published:** 2017-07-10

**Authors:** Kharleezelle J. Moendeg, Jose Ma M. Angeles, Ryo Nakao, Lydia R. Leonardo, Ian Kendrich C. Fontanilla, Yasuyuki Goto, Masashi Kirinoki, Elena A. Villacorte, Pilarita T. Rivera, Noboru Inoue, Yuichi Chigusa, Shin-ichiro Kawazu

**Affiliations:** 1 National Research Center for Protozoan Diseases, Obihiro University of Agriculture and Veterinary Medicine, Obihiro, Hokkaido, Japan; 2 Department of Biology, School of Science and Engineering, Ateneo de Manila University, Quezon City, Manila, Philippines; 3 Laboratory of Parasitology, Department of Disease Control, Graduate School of Veterinary Medicine, Hokkaido University, Sapporo, Hokkaido, Japan; 4 Department of Parasitology, College of Public Health, University of the Philippines Manila, Philippines; 5 Institute of Biology, University of the Philippines Diliman, Quezon City, Manila, Philippines; 6 Laboratory of Molecular Immunology, Department of Animal Resource Sciences, Graduate School of Agricultural and Life Sciences, The University of Tokyo, Tokyo, Japan; 7 Department of Tropical Medicine and Parasitology, Dokkyo Medical University School of Medicine, Tochigi, Japan; University of Melbourne, AUSTRALIA

## Abstract

**Background:**

Microsatellites have been found to be useful in determining genetic diversities of various medically-important parasites which can be used as basis for an effective disease management and control program. In Asia and Africa, the identification of different geographical strains of *Schistosoma japonicum*, *S*. *haematobium* and *S*. *mansoni* as determined through microsatellites could pave the way for a better understanding of the transmission epidemiology of the parasite. Thus, the present study aims to apply microsatellite markers in analyzing the populations of *S*. *japonicum* from different endemic areas in the Philippines for possible strain differentiation.

**Methodology/ Principal findings:**

Experimental mice were infected using the cercariae of *S*. *japonicum* collected from infected *Oncomelania hupensis quadrasi* snails in seven endemic municipalities. Adult worms were harvested from infected mice after 45 days of infection and their DNA analyzed against ten previously characterized microsatellite loci. High genetic diversity was observed in areas with high endemicity. The degree of genetic differentiation of the parasite population between endemic areas varies. Geographical separation was considered as one of the factors accounting for the observed difference between populations. Two subgroups have been observed in one of the study sites, suggesting that co-infection with several genotypes of the parasite might be present in the population. Clustering analysis showed no particular spatial structuring between parasite populations from different endemic areas. This result could possibly suggest varying degrees of effects of the ongoing control programs and the existing gene flow in the populations, which might be attributed to migration and active movement of infected hosts from one endemic area to another.

**Conclusions/ Significance:**

Based on the results of the study, it is reasonable to conclude that genetic diversity could be one possible criterion to assess the infection status in highly endemic areas. Genetic surveillance using microsatellites is therefore important to predict the ongoing gene flow and degree of genetic diversity, which indirectly reflects the success of the control program in schistosomiasis-endemic areas.

## Author summary

Schistosomiasis is one of the important neglected tropical diseases endemic in 78 countries throughout the world. The disease is caused by the parasitic worms known as schistosomes. In the Philippines, *S*. *japonicum* is the causative agent of the disease. The prevalence of the disease varies in endemic areas, suggesting that the parasite populations might differ in their genetic composition and infectivity to the human host. In this study, DNA samples of adult worms from seven endemic municipalities were analyzed. Characterization of *S*. *japonicum* samples in different endemic sites with varying prevalence provides information on the genetic diversity of the parasite. Results of this study showed that samples in high prevalence endemic areas like Irosin, Catarman and Socorro were genetically diverse as compared to other areas. Information on parasite genetic diversity is therefore important in planning disease control strategies. The results suggest ongoing parasite transmission across geographic endemic areas which should be monitored and used as reference for genetic diversity of the schistosomes attributed to geographic areas, thus a safeguarding precaution should be implemented to ensure localized elimination of the disease.

## Introduction

Schistosomiasis is one of the most important neglected tropical diseases affecting almost 240 million people throughout the world with more than 700 million considered to be at risk of infection [1]. Five species of schistosomes are known to cause human infection: *Schistosoma haematobium*, *S*. *mansoni*, *S*. *mekongi*, *S*. *intercalatum*, and *S*. *japonicum*. Among these species, *S*. *japonicum* is considered the most virulent because of the larger number of eggs it can produce as compared to other species, causing severe disease pathology. In addition, the zoonotic nature of *S*. *japonicum* contributes to increased disease transmission, making schistosomiasis control more difficult [2, 3].

Microsatellite markers have recently been used in determining *S*. *japonicum* genetic diversity and in estimating the levels of gene flow in the population. Previous studies have recommended the use of microsatellite markers to determine schistosome genetic diversity because of their codominant expression and their ability to serve as neutral markers [4, 5]. The easy observation of heterozygosity and the reasonable number of alleles per polymorphic locus in the samples make microsatellite analysis a more powerful tool in genetic studies than the use of rapid amplified polymorphic DNA (RAPD) and mitochondrial DNA [6]. Studying the genetic variation of *S*. *japonicum* populations provides an opportunity to link some genotypes associated with disease prevalence, which can then be used in formulating effective control measures. Previous studies using microsatellite markers suggested that the prevalence of *S*. *haematobium* and *S*. *mansoni* infections could be closely related with the parasite’s genetic variation. High prevalence of infection was observed in those areas with high genetic diversity and low prevalence in those areas with low genetic diversity [7, 8]. This information makes DNA microsatellites useful in population genetics studies.

In addition, microsatellite markers had been used in the identification of different geographical strains of *S*. *japonicum* in China [5]. Previous studies suggested that these markers could provide useful information for assessing the efficacy of mass drug administration (MDA) [9]. In the Philippines, schistosomiasis has shown considerable variations in the intensity and prevalence of the disease [10]. We hypothesized that the population genetic structure of schistosome parasites might partly contribute to these differences. The parasite population in each endemic area was therefore characterized for their genetic backgrounds using the microsatellite markers. The information found in this study can therefore provide basic information on the population genetic structure of *S*. *japonicum* in the Philippines that can be used as basis to evaluate and modify the widely used current control strategies for human schistosomiasis. For example, cognizant of the genetic types of the parasites, interventions can be modified so as to address differences in disease prevalence.

## Materials and methods

### Ethics statement

Animal experiments done in this study were conducted according to ethical guidelines for the use of animal samples permitted by the University of the Philippines Manila Institutional Animal Care and Use Committee (2011–009), as well as by Obihiro University of Agriculture and Veterinary Medicine (Permit No. 28–30). Infected mice were anesthesized using isoflurane before they were sacrificed. Perfusion method with normal saline solution was done to collect the adult *S*. *japonicum*.

### Parasite samples

**Fig 1:**
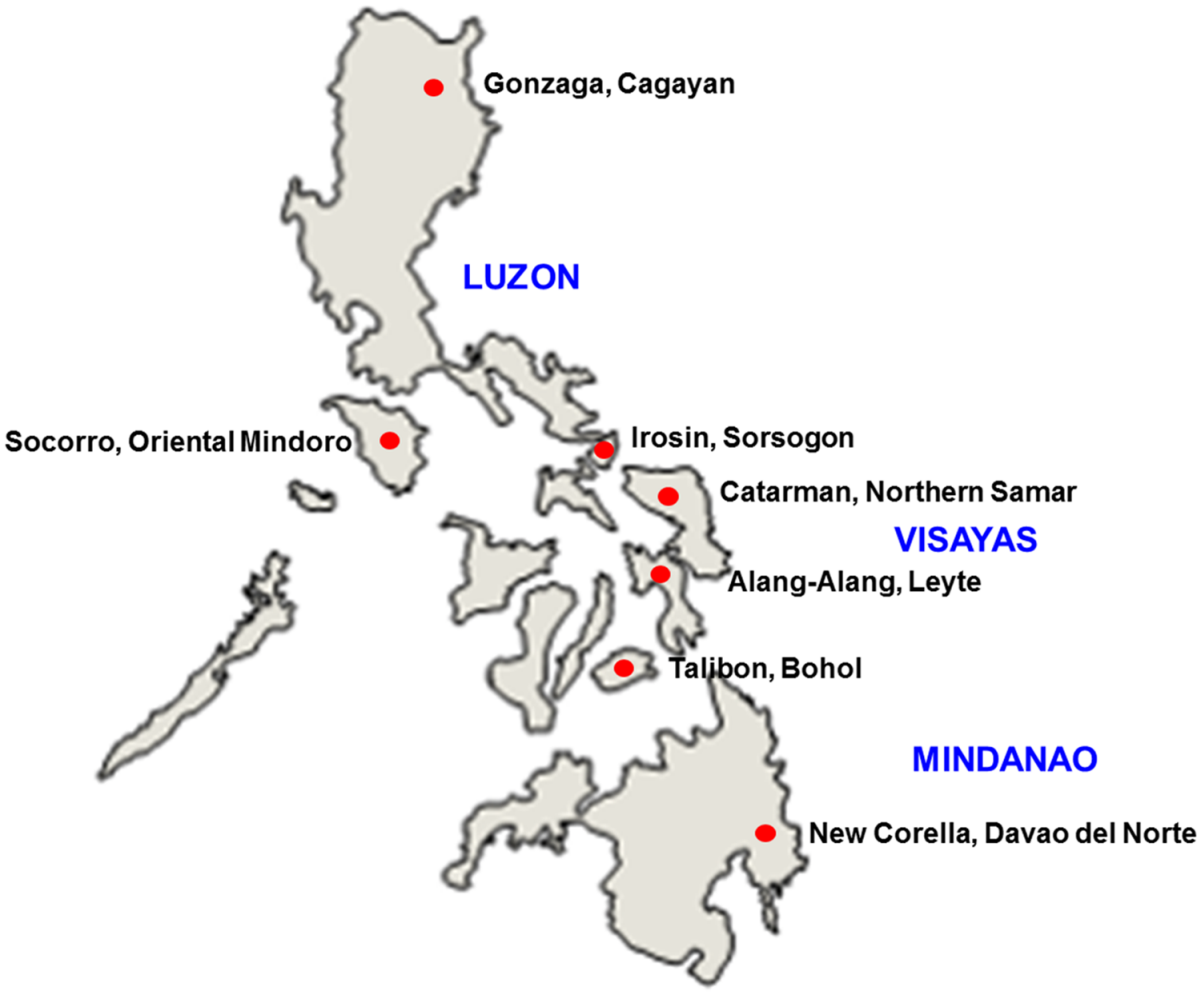
Study areas. The map shows the seven endemic municipalities in the Philippines included in this study. They are clustered into three major island groups Luzon, Visayas and Mindanao.

The snail intermediate host, *O*. *h*. *quadrasi*, were collected from seven municipalities (in seven provinces) in 2013 to 2015 where the disease is endemic, namely Catarman (in Northern Samar), Gonzaga (in Cagayan Province), New Corella (in Davao del Norte), Irosin (in Sorsogon), Talibon (in Bohol), Alang-Alang (in Leyte) and Socorro (in Oriental Mindoro) (Fig 1). The snails were crushed between glass slides to examine the presence of cercariae under the microscope. The cercariae were then pooled separately for each endemic municipality and used for mice infection. Ten BALB/c mice were infected percutaneously with 50 cercariae from each municipality. The infected mice were sacrificed six weeks after the infection, and the adult worms were collected from their mesenteric veins and washed with saline for DNA extraction.

### DNA extraction

Genomic DNA was extracted from individual male and female adult worms using the DNeasy Blood & Tissue Kit (QIAGEN, Japan) following the manufacturers’ protocol. Table 1 showed the total number of DNA samples tested in each endemic municipality.

**Table 1:** Population genetic analysis of *S*. *japonicum* from seven endemic municipalities in the Philippines.

Population	Sample size	A^a^	A^b^	Gene diversity (He) ^c^	Ho ^d^	F_IS_ ^e^
Catarman (Northern Samar)	32	8.300	4.500	0.727	0.842	0.012
Gonzaga (Cagayan)	21	6.700	3.800	0.605	0.646	0.161
New Corella (Davao del Norte)	25	5.000	3.460	0.587	0.750	-0.176
Irosin (Sorsogon)	45	11.500	4.630	0.694	0.725	0.239
Talibon (Bohol)	25	3.800	2.920	0.566	0.681	0.197
Alang-Alang (Leyte)	16	2.900	2.570	0.495	0.811	0.082
Socorro (Oriental Mindoro)	22	6.800	4.280	0.677	0.844	0.059

^a^ mean number of alleles

^b^ mean number of alleles corrected for sample size

^c^ gene diversity indices (expected heterozygosity)

^d^ observed heterozygosity

^e^ population-specific inbreeding coefficient values

### PCR amplification

PCR amplifications were performed using Veriti 96-well Thermal Cycler (Applied Biosystems, Carlsbad, CA). Amplifications were performed in 10 μl reactions containing 1 μl of 10X PCR buffer, 0.4 μl of 1.5 mM MgCl_2_, 0.2 μl of 2.5 mM dNTP, 0.2 μl each of 10 pmol/μl primer, 0.1 μl of 5 U/μl Taq DNA polymerase (Takara, Otsu, Japan), and 1 μl of template DNA. The conditions for thermal cycling were as follows: 5 minutes at 94°C, followed by 30 cycles of 1 minute at 94°C, 1 minute at locus-specific temperature, 1 minute at 72°C, with a final extension at 72°C for 10 minutes [11].

### Microsatellite genotyping

The DNA of each individual *S*. *japonicum* worm was genotyped using the previously characterized microsatellite loci RRPS, M5A, TS2, MPA, 2AAA, J5, SJP1, SJP5, SJP6, and SJP9 [11, 12]. In our study, we have screened twenty microsatellite markers but among which only ten worked well. The 5’ end of the forward primer for each locus was fluorescently labeled with 6-FAM, VIC and Ned dyes. Different dyes were used for those loci with overlapping fragment size. Two μl of the PCR product with LIZ 600 labeled size standard (Applied Biosystems) was subjected to the 3500 ABI Prism Genetic Analyzer for fragment analysis assay. The allele sizes were determined using the Gene Mapper software version 4.0 (Applied Biosystems). In each run, *S*. *japonicum* sample from Gonzaga, Cagayan, which has good DNA volume and concentration, served as the reference genotype for which the microsatellite sizes for the 10 loci had been determined by sequencing. *S*. *japonicum* Yamanashi strain (Japanese isolate) was also genotyped as a control group to confirm that the microsatellite markers could differentiate between samples from different origin. A total of 201 DNA samples were tested; however, only 186 were successfully genotyped due to poor DNA quality.

### Data analysis

For each population, the genetic diversity was examined by calculating the number of alleles using rarefaction analysis. Expected heterozygosity (gene diversity) (He) and observed heterozygosity (Ho) were determined using the GenAlEx 6.5 software [13]. Rarefaction analysis was performed to make the alleles comparable in the population. Genetic differentiation was determined using Wright’s F-statistics (Fst) in Arlequin, and the significance of the Fst values was tested at p value <0.05 [14]. The following qualitative guidelines were used for the interpretation of Fst genetic differentiation: 0–0.05 (little), 0.05–0.15 (moderate), 0.15–0.25 (great), and >0.25 indicate (very great genetic differentiation) [5]. The Analysis of Molecular Variance (AMOVA) was used to partition the genetic variation within and among populations using the software Arlequin version 3.5. The inbreeding coefficient (F_IS_), which measures the extent of nonrandom mating, was computed in the study. Nonrandom mating occurs when there is inbreeding.

Principal Coordinate Analysis (PCoA) was done to determine the clustering pattern of *S*. *japonicum* population based on their genetic distance using GenAlEx 6.5. Cercariae derived from a snail infected with only a single miracidium is assumed to be genetically identical. Hence, duplicate multi locus genotypes in a population are a consequence of clonal replication within snails [12, 15]. The presence of duplicate multilocus genotypes in adult worms was identified as one possible source of bias [15]. Duplicate multilocus genotype (MLG) was therefore removed, leaving a single representative of each in the dataset. Recent studies revealed that removal of clones in the dataset improved the assignment and clustering pattern of *S*. *japonicum* population [15]. The GENECLASS software 2.0 was used to identify migrant individuals [16].

To visualize relationships among populations, a Neighbor-joining tree was constructed based on F_ST_ genetic distance using 100 bootstrap replications in POPTREE2 [17]. The F_ST_ is one of the well-known parameters used in measuring genetic differentiation between populations using microsatellite data [18]. The *S*. *japonicum* Yamanashi strain was used as an outgroup.

### Accession numbers

Sequences of microsatellite loci reported here have been deposited in GenBank with the following accession numbers, RRPS (U22167), M5A (AF244896), TS2 (AF244896), MPA (U11895), 2AAA (M32280), J5 (M26212), SJP1 (EU262604), SJP5 (EU262608), SJP6 (EU262609) and SJP9 (EU262612).

## Results

### Genetic diversity

A total of 186 individual *S*. *japonicum* worms collected from seven endemic municipalities were analyzed. Highest gene diversity indices (He) was observed in Catarman (0.727) followed by Irosin (0.694), Socorro (0.677), and Gonzaga (0.605) while the lowest was in Alang-Alang (0.495). Those from New Corella (0.587) and Talibon (0.566) were comparable. Similarly, allelic richness after sample size correction was highest in Irosin (4.630) followed by Catarman (4.500) and Socorro (4.280) and lowest in Alang-Alang (2.570) followed by Talibon (2.920) (Table 1).

Population-specific inbreeding coefficient was determined in this study to measure the extent of nonrandom mating. Highest inbreeding coefficient values (F_IS_) was observed in Irosin (0.239) while the lowest was in Catarman (0.012) (Table 1). The lowest inbreeding coefficient values in Catarman may be related to the increased heterozygosity in this area. Inbreeding increases the homozygosity of the alleles. The pairwise F_ST_ values ranged from 0.019 to 0.0188, indicating varied levels of pairwise population genetic differentiation (Table 2). Great genetic differentiation was observed in the New Corella samples. The AMOVA showed that greater genetic variation in the samples occurred within the population (91.95%) rather than among populations (8.05%) (Table 3).

**Table 2:** Pairwise genetic differentiation (F_ST_) among populations based on 10 microsatellite loci ^a^.

Population	Catarman	Gonzaga	New Corella	Irosin	Talibon	Alang-Alang	Socorro
Catarman							
Gonzaga	0.093						
New Corella	0.141	0.168					
Irosin	0.060	0.068	0.145				
Talibon	0.081	0.062	0.162	0.042			
Alang-Alang	0.084	0.113	0.188	0.028	0.057		
Socorro	0.071	0.081	0.156	0.019	0.058	0.049	

^a^ All p values are significant below the level of 0.05 based on 100 permutations.

**Table 3:** Analysis of molecular variance (AMOVA) for *S*. *japonicum* population from different endemic municipalities.

Source of variation	Degrees of freedom	Sum of squares	Variance components	Percentage of variation (%) ^a^
Among population	6	118.129	0.33814	8.05
Within populations	331	1277.708	3.86014	91.95

^a^ Significance test (1023 permutations), p value <0.001

### Population structure

**Fig 2:**
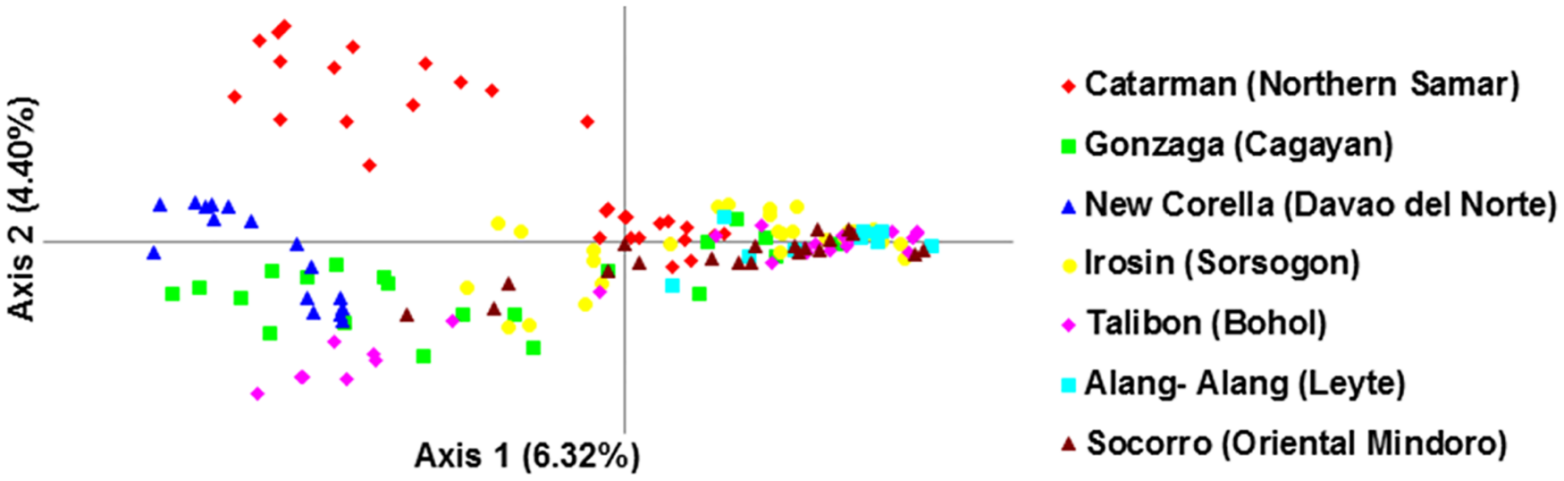
A two-dimensional plot of the Principal Coordinate Analysis (PCoA) of microsatellite data showing the clustering of *Schistosoma japonicum* populations after removal of duplicate genotypes. The proportion of variation is explained by each axis in parenthesis.

The PCoA showed no particular geographical structuring among the *S*. *japonicum* populations (Fig 2). The neighbor-joining tree method showed clustering of the samples into two groups. Populations from Catarman, New Corella, Gonzaga and Talibon grouped together (86% NJ bootstraps), whereas populations from Alang-Alang Socorro and Irosin belong to a separate cluster (61% NJ bootstraps) (S1 Fig). However, there was no correlation between the clustering of populations and their geographic distribution as shown in the neighbor joining tree (S1 Fig). These findings further support the results of the PCoA (Fig 2), suggesting the existing gene flow in the population. Two subgroups were observed in Catarman (Northern Samar) using the PCoA analysis (Fig 2). The presence of two subgroups in the Catarman samples may account for the high genetic variation within population (Table 1). Six samples, namely 3 from Gonzaga, 2 from Irosin and 1 from New Corella, were identified by GENECLASS as migrants (Table 4). These individuals showed a probability below 0.05.

**Table 4:** Results of migrant detection analysis in GENECLASS ^a^.

Sample (individuals)	GeneClass migrant likelihood ratio (L_h_/L_max_) p<0.05	Catarman	Gonzaga	New Corella	Irosin	Talibon	Alang-Alang	Socorro
Gonzaga	2.378	**25.119**	27.497	28.232	26.467	25.486	30.796	28.012
Gonzaga	0.692	19.157	7.410	22.428	21.506	**6.718**	25.575	24.229
Gonzaga	1.052	15.990	5.134	18.335	16.199	**4.082**	21.891	18.183
New Corella	5.706	**15.072**	21.517	20.779	21.082	22.944	21.800	20.741
Irosin	1.850	18.679	**16.293**	23.914	18.143	18.931	28.000	26.432
Irosin	0.371	**15.691**	30.700	24.214	16.062	25.013	22.569	20.470

^a^ The most likely source population for each individual is shown in bold.

## Discussion

In this study, based on the hypothesis that the population genetic structure of *S*. *japonicum* might explain the variations in the intensity and prevalence of schistosomiasis in the Philippines, the genetic polymorphism of the parasite population from different endemic areas was examined. A large number of different alleles were observed in the samples examined, especially in Irosin, Catarman and Socorro where high prevalence of infections was reported [10] (S1 Table). There is a greater potential for these populations to possess the alleles responsible for the parasite infectivity, causing high infection [7, 19]. These findings were in agreement with that of previous studies where the prevalence of infection was directly proportional to the number of alleles [7, 8, 22]. This situation somehow follows a general pattern in our current study where high prevalence of infection either in humans and snail hosts was observed in those areas with high allelic richness while low or zero prevalence in those areas with low allele numbers (S1 Table). However, this is in contrast to our results in Alang-Alang where low number of different alleles has been observed where high prevalence of the infection was also reported. This could be due to the prolonged utilization of praziquantel from the annual MDA since Leyte has been one of the oldest endemic foci in the Philippines. To confirm such findings the effect of drug selective pressure brought by praziquantel on the parasite genetic diversity should be analysed. It has been known in other parasitic infections such as malaria that selective drug pressure brought by extensive drug use can lead to a reduction in genetic diversity of the parasite [20, 21]. Currently, there are no microsatellite markers that can be linked with parasite infectivity. The alleles that might contribute to the high infection rate might be present in those area with high prevalence, however further studies should be done to confirm this.

In this study, it should be noted that the prevalence data presented in S1 Table was collected from 2013 to 2015 while, our samples obtained from Talibon (Bohol) were collected prior to this period. Bohol is considered as a near-elimination area based on the absence of human cases for many years now. However, the presence of infection in water buffaloes continues to indicate an ongoing transmission even if there are no more human cases (S1 Table). Hence, the possibility of human infection is always present.

Among the seven endemic municipalities analyzed, the Catarman samples showed the highest gene diversity indices (Table 1). Catarman has been reported with high prevalence of infection both in humans and snail intermediate hosts (S1 Table). In addition, this study also revealed that water buffaloes and dogs in this municipality had high prevalence of infection (S1 Table). High infection rate in humans and animal hosts will then increase the probability of snail infection. A study by Rudge *et al*. (2008) in the Philippines using *S*. *japonicum* larval stages, found high levels of parasite gene flow between humans and dogs suggesting strongly the frequent transmission of *S*. *japonicum* infection across host species and between villages [23]. Furthermore, the role of animals in disease transmission was further supported by a population genetics study in China, where *S*. *japonicum* from cattle showed high genetic diversity in the marshland areas, whereas parasites from humans and dogs were more diverse in the hilly region [24, 25]. These previous studies have therefore demonstrated the contribution of animal host species in the genetic diversity, and gene flow pattern of the parasite. Thus, the zoonotic nature of *S*. *japonicum* infecting animals should be seriously considered in the increased disease transmission [26, 27, 28]. Currently, we are now performing direct genotyping of stool-derived eggs collected from humans and animals particularly in water buffaloes and dogs using microsatellites. We will measure the infection intensities in humans and animals together with the parasite’s genetic variation in our ongoing study. Moreover, in this study we chose to use adult worms isolated from mice experimentally infected with snail-derived cercariae for genotyping. This is because adult worms can generally provide DNA with higher quality and quantity suitable for genotyping than snail-derived cercariae or eggs in stool samples. However, fitness of parasites to mice may serve as a bias to the genetically diverse population, leading to bottlenecking of genotypes [29, 30]. The *S*. *japonicum* cercariae shed from infected snails collected from endemic areas will also be analyzed in our future studies. Because there are several studies on parasite’s population genetics by using cercariae-derived samples [23, 24, 25], such the direct genotyping may be feasible and provide an advantage to skip in vivo passage.

The level of genetic differentiation differs between endemic areas. Great genetic differentiations were observed in the New Corella samples than those from other endemic sites (Table 2). The large geographical distance separating New Corella from other endemic sites could possibly limit the contact between the hosts, eventually resulting to high genetic differentiation in this municipality. New Corella is located in Southern Mindanao and is expected to be more genetically differentiated because of its geographic location (Fig 1). As seen in Fig 1, New Corella is the farthest of the endemic municipalities being separated by a wide distance from other endemic municipalities. Previous studies showed that the high genetic differentiation observed among peripheral populations such as those of New Corella can be explained by their strong spatial isolation [31].

Furthermore, the possibility of the snail hosts influencing the genetic variation of the parasite population should also be taken into consideration. Presently, there is no study using microsatellites on the genetic variation of the snail population in the sampling areas. However, previous studies on mitochondrial DNA had provided some insights into the genetic variation of the snail population of *S*. *japonicum*, and suggested that examination of naturally infected snails may exhibit co-evolutionary relationships with their parasites [32]. Thus, a snail population may reflect the population genetic parameters of their parasites [33, 34]. Nevertheless, it is worth mentioning that these previous studies on the genetic diversity of *Oncomelania* populations are based on mitochondrial markers. Hence, future studies using microsatellites on the snail populations from each endemic area is essential to obtain results which can be analyzed together with those of *S*. *japonicum* population.

The genetic variation observed using AMOVA was greater within each *S*. *japonicum* population (91.95%) than the variation among the populations (8.05%) (Table 3). This might be due to the snails being infected by genetically different cercariae having multiple genotypes within the endemic areas [7, 22]. Mixing of infected snails and of their parasites brought about by flooding may explain the higher genetic variation within the population [5, 9]. Also, the continuous rainfall and subsequent floods in these endemic areas might facilitate host-parasite contact, exposing people and animals to contaminated waters that result to higher infection. Thus, people and animals moving from one village to another to escape flooding, take advantage of employment opportunities and there is also animal trade where water buffaloes are exported to other areas could facilitate parasite transmission, contributing to high genetic variation within each endemic area. Another reason could be due to the snail sampling being done in three villages for each endemic municipality where a high village-level variation might exist. Genetic variance within population was accounted for most of the genetic diversity of *S*. *japonicum* population in endemic provinces in China [5].

*S*. *japonicum* samples obtained from different endemic areas did not form a particular spatial structuring. The lack of geographical structuring suggests that there is still an ongoing gene flow among the *S*. *japonicum* populations in all the study areas despite execution of control measures [22]. These findings might imply that there is a continuing transmission of *S*. *japonicum* across geographic areas, and therefore reflect the inadequate effect of MDA implementation. The current national control strategy for schistosomiasis in the Philippines is annual MDA using 40 mg/kg of praziquantel in all schistosomiasis-endemic villages including the sampling areas. However, the compliance rate was reported to be <50% [35, 36].

The ongoing gene flow in the populations might be attributed to migration and movement of infected hosts as also suggested otherwise by previous studies done on *S*. *mansoni* [22]. The infected hosts could therefore serve as means of allele dispersal in endemic sites. Therefore, the existence of gene flow among the schistosome populations might increase the opportunity for the spread of alleles conferring parasite traits such as infectivity, virulence and drug resistance [8, 22].

Two subgroups were observed in the Catarman samples using the PCoA analysis (Fig 2), indicating that co-infection with several genotypes of the parasite might be infecting the hosts in this endemic site. Catarman is surrounded by other endemic areas such as Leyte, Negros Occidental, Bohol in the Visayas, so the possibility of intermixing of the parasite is very high leading to high genetic variation within the area. A higher transmission and infection success is expected to occur more in mixed parasite genotypes than in single-genotype infection as reported in previous studies [37]. There might be a decrease in the effectiveness of the host immune system to cope with the infection due to the simultaneous attack of the parasite with different genotypes leading to a higher infection success [37]. Furthermore, the high genetic diversity in Catarman may be explained by the lowest inbreeding coefficient values. Inbreeding increases the similarity of the alleles in the parasite population. Previous studies suggested that co-infection by multiple genotypes decreases the possibility of inbreeding [37].

Some parasite populations in Gonzaga, New Corella and Irosin were identified as migrants using Gene Class 2.0 software (Table 4). Gonzaga has just been identified as a new endemic focus at the start of the 21^st^ century [38], and it is presumed that some parasite populations are introduced in this area from Talibon and Catarman. Infected people or animals might have moved from these areas and started the disease transmission in Gonzaga. The theory proposed for the emergence of schistosomiasis in Gonzaga was based on the history of a big geothermal project by PNOC (Philippine National Oil Company) in Gonzaga that recruited workers from the Visayas and Mindanao (Fig 1). The movement of people from these endemic areas into Gonzaga brought in cases, and with the presence of snail hosts in the area, the emergence of the disease became just a matter of time [38]. Interestingly, migrants detected in Irosin were rooted from Gonzaga, further providing evidence for the continuous transmission flow of the parasite among the endemic municipalities in the Philippines (Table 4). Migrant detection in the study supports the clustering analysis using the neighbor joining method wherein the population, including those individuals that were considered to be migrants, clustered together with their source population; for instance, Gonzaga received migrants from Talibon, which clustered together in the NJ tree (S1 Fig).

Genetic diversity found in this study among the parasite population in each endemic site in the Philippines is vital in the parasites’ ability to survive the effect of selective pressures such as those brought by drug treatment. At the same time, selection pressures increase the frequency of favorable alleles across all populations [4]. In this sense, the present finding of high diversity among the parasite populations imply that the MDA with praziquantel has varying degree of impact in interrupting the parasite’s life cycle. Aside from looking at several factors that can contribute to possible treatment failures including low compliance and the quality of the drugs used, it is also important that the effects of MDA be monitored on schistosome populations for its genetic background. This can be done by using microsatellite markers to measure the genetic diversity parameters which include the allelic richness and the heterozygosity of the alleles in the parasite population before and after MDA implementation. Alleles contributing to the severity of the disease such as the ones responsible for the fecundity and survival of *S*. *japonicum* inside the host should be identified and need to be further studied.

In conclusion, the use of microsatellites in this study has shown that there is an ongoing gene flow among the *S*. *japonicum* population from different endemic areas, indicating the active movement of infected humans and animals from one endemic area to another. Aside from the control programs being implemented in each endemic area, an effective surveillance to monitoring these movements in humans and animals in each endemic site should be in place. Thus, a better cooperation between the medical and veterinary sectors would be highly recommended to ensure a strengthened control program for schistosomiasis. In addition, the diversity will indirectly explain the varying degree of the effects of the ongoing control programs done in these endemic areas. A regular MDA should be implemented and monitored regularly for its efficacy in endemic areas. Considering that only 10 microsatellite markers were analyzed in this study for determining genetic diversity and gene flow of the parasite, we therefore recommend the use of additional highly polymorphic microsatellite markers not only for *S*. *japonicum*, but for *S*. *mansoni* and *S*. *haematobium* to be used in future studies for more precise analysis.

## Supporting information

S1 Table Prevalence of *S*. *japonicum* infection in snails, humans and animal hosts from different endemic areas in the Philippines (2013–2015). (DOC)

S1 Fig Neighbor-joining tree based on F_ST_ genetic distance showing the phylogenetic relationships of *S*. *japonicum* originating from each endemic site. Values on nodes are percentage bootstrap supports based on 100 bootstrap samples. Scale bar represents F_ST_ genetic distance of 0.05.
(TIF)

S1 Dataset Microsatellite data showing the allele sizes of the samples tested using ten markers. (XLSX)
