# Supplementary material for: Geographic strain differentiation of Schistosoma japonicum in the Philippines using microsatellite markers
Source: PLoS Negl Trop Dis. 2017 Jul 10;11(7):e0005749. doi: 10.1371/journal.pntd.0005749 (PMC5519200; doi:10.1371/journal.pntd.0005749)
Supplement: S1 Table — (DOC) [file pntd.0005749.s001.doc]

**Supporting Information**

**S1 Table. Prevalence of *S. japonicum* infection in snails, humans and animal hosts from different endemic areas in the Philippines (2013-2015).**

| Animal prevalence rate % (N) ^c^ | Human prevalence rate % (N) ^a,b^ | Snail infection rate % (N) ^a^ | Endemic areas |
| --- | --- | --- | --- |
|  | 7.92 (442) | 1.2 (500) | Socorro (Oriental Mindoro) |
|  | 7.10 (183) | 0.57 (1588) | Alang- Alang (Leyte) |
|  | 6.73 (202) | 1.25 (1123) | Irosin (Sorsogon) |
| 7.14 (28) water buffaloes | 0 (143) | 0 (300) | Talibon (Bohol) |
|  | 1.66 (421) | 1.1 (1500) | Gonzaga (Cagayan) |
|  | 0 (187) | 0.5 (1810) | New Corella (Davao del Norte) |
| 38.78 (49) water buffaloes, 50.00 (16) dogs | 10.06 (358) | 12.77 (329) | Catarman (Northern Samar) |

^a^ Leonardo et al., 2016

^b^ human prevalence determined by Kato-Katz technique

c animal prevalence determined by stool PCR
