# Supplementary figures and images for: Geographic strain differentiation of Schistosoma japonicum in the Philippines using microsatellite markers
Source: PLoS Negl Trop Dis. 2017 Jul 10;11(7):e0005749. doi: 10.1371/journal.pntd.0005749 (PMC5519200; doi:10.1371/journal.pntd.0005749)

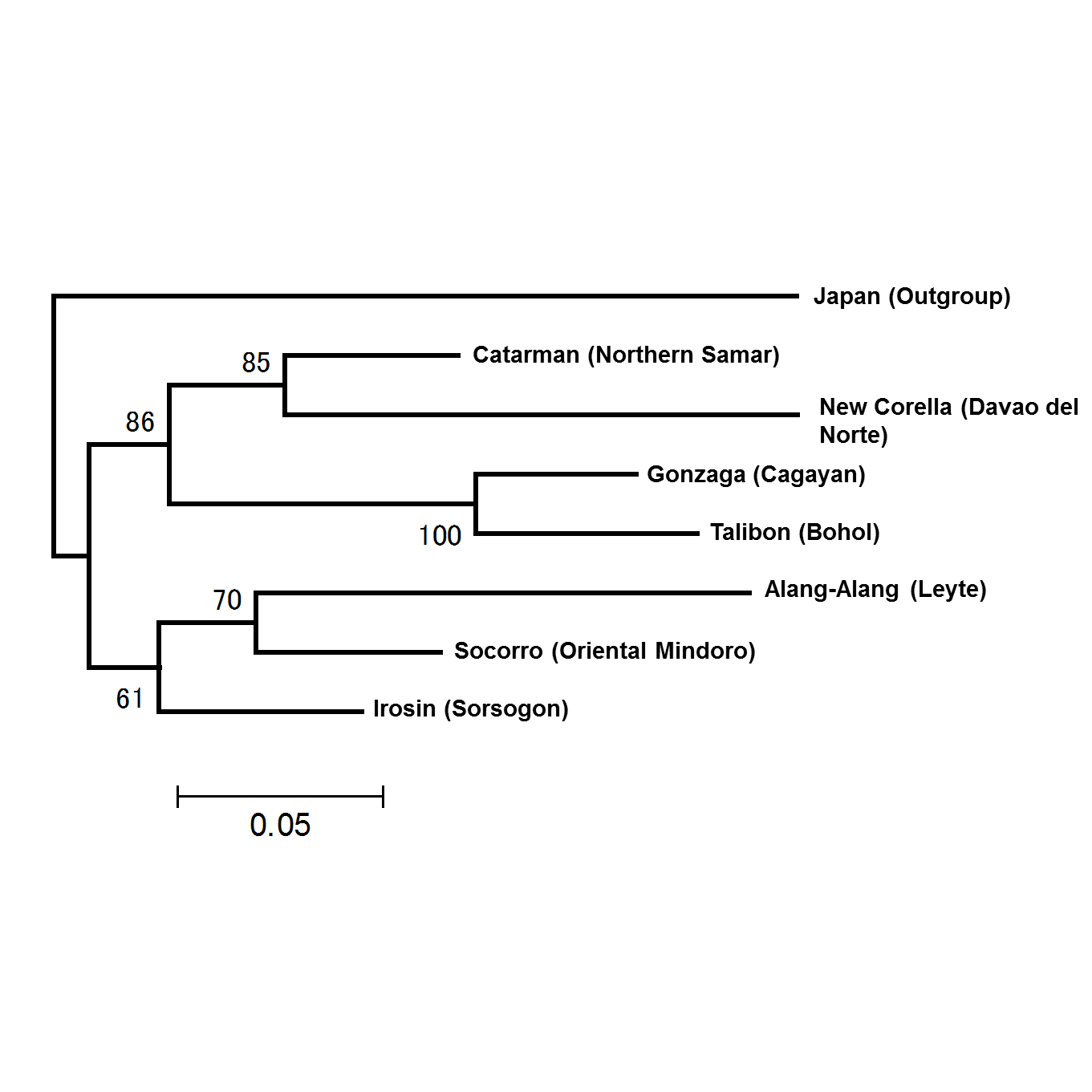

Supplement: S1 Fig — Values on nodes are percentage bootstrap supports based on 100 bootstrap samples. Scale bar represents FST genetic distance of 0.05. (TIF) [file pntd.0005749.s002.tif]
